# Kisspeptin neurones in the posterodorsal medial amygdala modulate sexual partner preference and anxiety in male mice

**DOI:** 10.1111/jne.12572

**Published:** 2018-02-27

**Authors:** D. A. Adekunbi, X. F. Li, G. Lass, K. Shetty, O. A. Adegoke, S. H. Yeo, W. H. Colledge, S. L. Lightman, K. T. O'Byrne

**Affiliations:** ^1^ Division of Women's Health Faculty of Life Sciences and Medicine King's College London London UK; ^2^ Department of Physiology College of Medicine University of Lagos Lagos Nigeria; ^3^ Reproductive Physiology Group Department of Physiology, Development and Neuroscience University of Cambridge Cambridge UK; ^4^ Henry Wellcome Laboratory for Integrative Neuroscience and Endocrinology University of Bristol Bristol UK

**Keywords:** amygdala, anxiety, kisspeptin, mice, partner preference, sexual behaviour

## Abstract

The posterodorsal medial amygdala (MePD) is a neural site in the limbic brain involved in regulating emotional and sexual behaviours. There is, however, limited information available on the specific neuronal cell type in the MePD functionally mediating these behaviours in rodents. The recent discovery of a significant kisspeptin neurone population in the MePD has raised interest in the possible role of kisspeptin and its cognate receptor in sexual behaviour. The present study therefore tested the hypothesis that the MePD kisspeptin neurone population is involved in regulating attraction towards opposite sex conspecifics, sexual behaviour, social interaction and the anxiety response by selectively stimulating these neurones using the novel pharmacosynthetic DREADDs (designer receptors exclusively activated by designer drugs) technique. Adult male Kiss‐Cre mice received bilateral stereotaxic injections of a stimulatory DREADD viral construct (AAV‐hSyn‐DIO‐hM_3_D(Gq)‐mCherry) targeted to the MePD, with subsequent activation by i.p. injection of clozapine‐*N*‐oxide (CNO). Socio‐sexual behaviours were assessed in a counter‐balanced fashion after i.p. injection of either saline or CNO (5 mg kg^‐1^). Selective activation of MePD kisspeptin neurones by CNO significantly increased the time spent by male mice in investigating an oestrous female, as well as the duration of social interaction. Additionally, after CNO injection, the mice appeared less anxious, as indicated by a longer exploratory time in the open arms of the elevated plus maze. However, levels of copulatory behaviour were comparable between CNO and saline‐treated controls. These data indicate that DREADD‐induced activation of MePD kisspeptin neurones enhances both sexual partner preference in males and social interaction and also decreases anxiety, suggesting a key role played by MePD kisspeptin in sexual motivation and social behaviour.

## 1 INTRODUCTION

The posterodorsal medial amygdala (MePD) has received considerable attention as part of a neurobiological network involved in sexual behaviour. This neural locus in the limbic system participates in interpreting olfactory chemosignals,^1^, ^2^, ^3^ genitosensorial stimulation^4^ and regulating social/sexual behaviour in rodents.^5^, ^6^, ^7^, ^8^ Sexual cues from females activate neurones within the MePD of male mice,^9^ which may be analogous to increased amygdala neuronal activity in humans exposed to arousing sexual images.^10^ The MePD is involved in sexual behaviours such as investigation and attraction towards opposite sex‐conspecifics.^11^ Preference towards an opposite sex odour was hampered by lesioning the MePD in mice,^12^ rats^13^ and hamsters.^14^, ^15^ The resultant deficit in mate preference as a result of MePD lesion was accompanied by a prolonged latency to mount or ejaculate,^15^ which suggests a critical role for the MePD in sexual motivation and copulatory behaviour. Additionally, the MePD is functionally related to fear and anxiety. The induction of c‐Fos has been reported in the MePD of rats subjected to anxiety using the elevated plus maze.^16^ Also, social defeat robustly activates the MePD,^17^ whereas its lesioning decreased playful fighting behaviour in rats.^18^ Collectively, these data indicate that the MePD serves an overlapping function integrating emotional and sexual behaviour.

Although lesion and immediate early gene expression studies have identified an important role played by the MePD in socio‐sexual behaviour, they are limited in clarifying the definitive function of the specific neuronal cell type involved and, as such, there is a lack of adequate information on how sexual cues processed within the MePD bring about the appropriate behaviour. The recent discovery of a significant kisspeptin neurone population in the MePD^19^ has raised interest in the probable role of kisspeptin and its cognate receptor in sexual behaviour. Kisspeptin receptor (*Kiss1r*) knockout male mice lack the characteristic preference for oestrous female odour,^20^ suggesting an essential role of kisspeptin signalling in mediating mate preference. We have recently shown the specificity of the MePD as a neural site for the role of kisspeptin in sexual behaviour, with micro‐infusion of kisspeptin into the MePD causing ex‐copula erections in male rats.^21^ On the other hand, little is known about the role of kisspeptin in the fear and anxiety response, although it was recently reported that habenula kisspeptin modulates fear in zebrafish.^22^ In rats, the role of kisspeptin on anxiety is controversial,^23^, ^24^ whereas it attenuated negative mood in humans.^25^

In the present study, we employed the pharmacosynthetic DREADDs (designer receptors exclusively activated by designer drugs) technique to selectively stimulate kisspeptin neurones in the MePD of Kiss‐Cre male mice aiming to investigate whether endogenous MePD kisspeptin signalling potentiates a preference for oestrous female mice, in addition to regulating sexual behaviour, the anxiety response and social interaction.

## 2 MATERIALS AND METHODS

### 2.1 Animals

Breeding pairs of Kiss‐Cre heterozygous transgenic mice^26^ weighing between 25 and 30 g were obtained from the Reproductive Physiology Group of University of Cambridge (Cambridge, UK). Litters from the breeding pairs were genotyped by polymerase chain reaction (PCR) analysis. Male mice heterozygous for the Kiss‐Cre transgene and experiencing normal pubertal development, as established by the age of pubertal onset, and as also confirmed to be sexually active, were included in the study. Mice were housed under a 12:12 hour light/dark cycle (lights on 07.00 hours) at 22 ± 2 °C and provided with food (standard maintenance diet; Special Dietary Services, Wittam, UK) and water ad libitum. All animal procedures were performed in accordance with the UK Home Office Regulations.

### 2.2 Stereotaxic injection of DREADD virus

Surgical procedures for stereotaxic injection of stimulatory Cre‐dependent DREADD viral construct (AAV‐hSyn‐DIO‐hM_3_D(Gq)‐mCherry, Serotype:5; University of North Carolina at Chapel Hill Vector Core, NC, USA) to express the hM_3_Dq‐DREADD in MePD Kiss1 neurones were performed under aseptic conditions with general anaesthesia induced by ketamine (Vetalar, 100 mg kg^‐1^, i.p.; Pfizer, Sandwich, UK) and xylazine (Rompun, 10 mg kg^‐1^, i.p.; Bayer, Leverkusen, Germany). Kiss‐Cre male mice (age 7‐8 weeks, n = 12) were secured in a David Kopf stereotaxic frame and small holes were drilled into the skull at a location above the MePD after a midline incision of the scalp. The stereotaxic injection coordinates used to target the MePD were obtained from the mouse brain atlas of Paxinos and Franklin^27^ (2.1 mm lateral, 1.64 mm posterior to bregma and 5.1 mm below the surface of the dura). Using a 2‐μL Hamilton micro syringe (Esslab, Essex, UK), 1 μL of AAV‐hSyn‐DIO‐hM_3_D(Gq)‐mCherry was injected bilaterally into the MePD over 10 minutes. The needle was left in position for a further 5 minutes and then removed slowly over 1 minute. After recovery from surgery, mice were left undisturbed for 4 weeks to allow the time‐course for effective and stable receptor expression.^28^

### 2.3 Behavioural tests

Behavioural tests include sexual partner preference, sexual behaviour, anxiety and social interaction tests. A cross‐over design was employed to test all mice with vehicle (saline) and clozapine‐N‐oxide (CNO; Tocris Bioscience, Bristol, UK). CNO was administered i.p. in saline as vehicle at a dose of 5 mg kg^‐1^.^29^ On the day of testing, half of the mice received vehicle or CNO injections, whereas the other half received injections in the reverse order on subsequent testing, leading to an animal being tested twice for a given behavioural test in a counter‐balanced order, with each test separated by 3‐5 days. The behavioural tests were designed and ordered to first access olfactory‐related processes such as sexual partner preference and social interaction, followed by sexual behaviour and a standard anxiety test. All behavioural tests were conducted in a dimly lit room between 12.00 and 14.00 hours, and tests commenced 30 minutes post vehicle or CNO injection to enable activation of hM_3_Dq receptor by CNO.^30^ Behavioural events were manually scored by 2 experimenters who were blind to the treatment.

#### 2.3.1 Sexual partner preference

Sexual partner preference testing was carried out as described by Dresroziers et al.^31^ and Angoa‐Perez et al.^32^ using a 3 chamber compartment as described below. A rectangular plexiglass cage (60 × 13 × 30 cm; Techniplast, Buguggiate, Italy) was divided into 3 equal compartments by an opaque partition with an opening (5 × 5 cm) at floor level. Mice were habituated to the 3‐compartment box for 10 minutes prior to the commencement of the test. Once testing began, an oestrous female mouse confirmed by vaginal cytology and gonadally‐intact male mouse housed in a wire mesh cup were randomly introduced into each of the lateral compartments of the box. The mesh cup does not permit physical contact and only allows visual, olfactory and vocal communications. The number of entries by the test mouse into each lateral compartment containing either the male or female conspecific, as well as the time spent actively sniffing or poking its nose near the holes of the mesh cup, was recorded over a 10‐minute test period. Preference score was determined by subtracting the time spent with the oestrous female from that spent with the male. A positive score indicates preference for female, whereas a negative score indicates preference for male.^33^

#### 2.3.2 Sexual behaviour

Sexual behaviour testing was conducted in a Techniplast cage (32 × 16 × 14 cm) with clean wood chip bedding. Males were given sexual experience by co‐habitation with receptive females for about 2 weeks before the test. On the day of the test, mice were habituated to the test arena for 10 minutes before introducing the oestrous female. The latency for mounting, intromission and ejaculation, as well as the number of mounts and intromissions, was recorded. The test was terminated once the mouse ejaculated or after 30 minutes of testing. If no sexual behaviour was displayed within the 30‐minute test period, the latency was scored as 1800 seconds. A mount was scored as the male climbing and grabbing the female from behind with both paws. Intromission was designated as vaginal penetration during mounting accompanied by pelvic thrusting, whereas ejaculation was scored as intromission with a longer lasting thrust resulting in the male immobilising and falling off the female followed by a period of disinterest in the female.^34^

#### 2.3.3 Anxiety‐like behaviour

The elevated plus maze (EPM) was used to assess anxiety. The EPM apparatus was made from plexiglass and consisted of 2 opposing open arms (30 × 5 cm) and 2 closed arms (30 × 5 cm, enclosed by 15 cm walls on each side). The central platform measures 5 × 5 cm and the apparatus was elevated 40 cm above the floor. The mouse was placed at the central platform facing the closed arm of the maze. Time spent in the open and closed arms, as well as the number of entries into each arm, was recorded during the 5‐minute period. An entry into the arm was defined as all 4 paws in the arm, whereas an exit was defined as at least 2 paws out of the arm. Anxiety index (AI) was determined from total activity on the EPM using the formula described by Cohen et al^35^ The AI score ranges from 0 to 1; a higher index indicates increased anxiety‐like behaviour.

#### 2.3.4 Social interaction

Test mice were singly housed for 1 hour in a holding room adjacent to the test room. At the commencement of the test, the test mouse and a same sex and strain juvenile conspecific (23‐28 days old)^36^ were placed simultaneously in the test arena, comprising a Techniplast cage (32 × 16 × 14 cm) with clean wood chip bedding. The total time spent sniffing, following, grooming and mounting the conspecific was recorded over a period of 5 minutes. The use of a juvenile mouse as a social stimulus should trigger less aggressive bouts in adult mice^37^ and limit confounding aggressive responses typical of adult male‐male interaction^38^ in the social repertoire.

### 2.4 Validation of injection site

Mice were anaesthetised with a lethal dose of ketamine and transcardially perfused with heparinised saline for 5 minutes, followed by 10 mL of ice‐cold 4% paraformaldehyde (PFA) in 0.1 mol L^‐1^  phosphate buffer (pH 7.4) for 15 minutes using a pump (Minipuls, Gilson, Villiers Le Bel, France). Brains were rapidly collected and postfixed sequentially at 4°C in 15% sucrose in 4% PFA and in 30% sucrose in 0.1M phosphate‐buffered saline until they sank. Afterwards, brains were snap‐frozen on dry ice and stored at −80°C until processing. Brains were cut into 30‐μm thick coronal sections using a cryostat (Bright Instrument Co., Luton, UK) and every third section was collected between −1.34 mm to −2.30 mm from the bregma. Sections were mounted on microscope slides, air‐dried and cover slipped with ProLong Antifade mounting medium (Molecular Probes, Inc. OR, USA). Precise injection sites were verified and evaluated and only animals expressing mCherry fluorescent protein unilaterally or bilaterally in the MePD were included in the behavioural analysis. Positive neurones expressing mCherry fluorescent protein throughout the MePD were quantified using an Axioskop 2 Plus microscope (Zeiss, Gottingen, Germany). The neuroanatomical landmarks bordering the MePD were determined using a reference guide from the mouse brain atlas.^27^ The number of mCherry positive neurones was counted in the MePD of each animal and the total number was used to calculate the group mean (mean ± SEM). Images were taken using Axioskop 2 Plus microscope (Zeiss) equipped with axiovision, version 4.7 (Zeiss).

### 2.5 Statistical analysis

Comparisons between vehicle and CNO‐induced DREADDs activation on behavioural events were made by subjecting data to Mann‐Whitney *U* test (Systat Software Inc., San Joses, CA, USA). Student's *t* test was used to compare the mean numbers of positive mCherry fluorescent neurones in the MePD from mice observed bilaterally with the 1 from mice observed unilaterally. Data are reported as the mean ± SEM. *P* < .05 was considered statistically significant.

## 3 RESULTS

### 3.1 Selective targeting of MePD Kiss1 neurones

**Figure 1:**
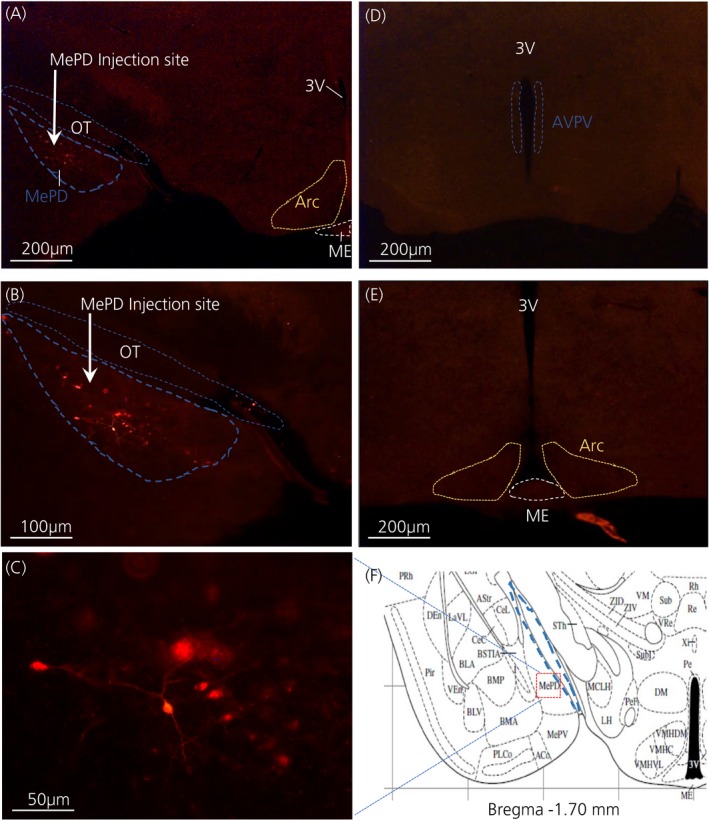
Expression of posterodorsal medial amygdala (MePD) kisspeptin neurones with hM3Dq‐mCherry in Kiss‐Cre mice. Coronal section shows red mCherry fluorescence positive neurones (blue line) in the MePD but not in the arcuate nucleus (ARC) (A) and the white arrow indicates the injection site of AAV‐hSyn‐DIO‐hM_3_D(Gq)‐mCherry into the MePD of Kiss‐Cre mice in the same section (B). Higher‐power view shows the MePD kisspeptin neurones tagged with mCherry (red fluorescence), which indicates hM3Dq receptor expressing kisspeptin neurones (C). The absence of red fluorescence in the anteroventral periventricular nucleus (AVPV) (blue dotted line) (D) and arcuate nucleus (ARC) (yellow dotted line) (E) shows the specificity of the viral contruct to MePD kisspeptin neurones. Schematic representation^27^ of MePD and its spatial relationship with the optic tract (blue dotted line) (F). ME, median eminence; OT, optic tract; 3V, third ventricle

The Cre‐dependent DREADDs confine to the Cre locus of targeted neural site^30^ and tagging the hM_3_Dq receptor with mCherry facilitates visibility under the fluorescence microscope. Analysis of the acquired images showed that 9 out of 12 mice receiving a stereotaxic injection of the hM_3_Dq viral construct displayed mCherry fluorescence signals localised in the MePD; bilateral (n = 5) and unilateral (n = 4). The mean numbers of mCherry fluorescent positive neurones in the MePD were 23.80 ± 5.26 for mice expressing mCherry bilaterally and 14.50 ± 4.57 for mice with unilateral mCherry expression. However, there was no statistically significant difference between the 2 groups (*P* > .05), which may be a result of variation in the number of mCherry positive neurones among the experimental animals. The expression of mCherry fluorescent protein only unilaterally in some animals may result from needle misplacement or blockade during surgery. Furthermore, analysis with a Mann‐Whitney *U* test indicated that there were no variation in behavioural outcome in mice with either bilateral or unilateral hM_3_Dq expression in the MePD; therefore, their data were pooled for further statistical analysis. Animals with misplaced injection sites, as defined by the absence of mCherry fluorescent protein in the MePD, had similar behavioural data with or without CNO treatment and some of their data provided in the sections below. A representative photomicrograph of a coronal brain section is shown in Figure 1.

### 3.2 Effect of CNO on sexual partner preference

**Figure 2:**
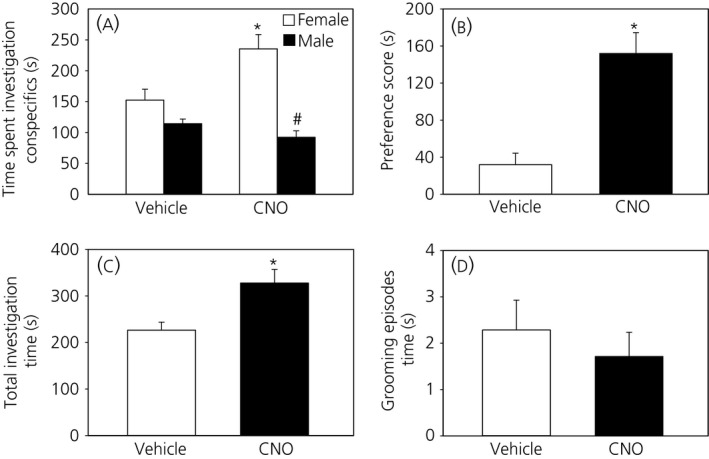
Effect of clozapine‐*N*‐oxide (CNO) on sexual partner preference. CNO (5 mg kg^‐1^; i.p.) resulted in a significant increase in the time spent investigating oestrous females compared to males, whereas, with vehicle (saline), there was only a tendency for increased investigation of oestrous female (A). The partner preference score for oestrous female was significantly increased following treament with CNO compared to vehicle (B). The overall investigatory time with both male and female conspecifics was significantly greater with CNO (C). Genital grooming episodes were not significantly differerent with or without CNO (D). *P < .05 vs vehicle, ^**#**^ *P* < .05 vs female in response to CNO (n = 9). The results are the mean ± SEM

When presented with a choice between an oestrous female and a male, test mice preferred the oestrous female rather than the male. The mate preference score was significantly higher following CNO treatment compared to vehicle and this indicates that DREADD‐induced activation of MePD Kiss1 neurones enhances sexual partner preference (*P* < .05) (Figure 2A,B). In mice with misplaced injection sites, which serve as a negative control, the preference score was comparable with or without CNO treatment (CNO; 57.0 ± 8.62 vs Saline; 50.3 ± 8.41 seconds). Meanwhile, the attraction of the male mice to the oestrous female did not reflect in the number of entries to the compartment containing the stimulus; there were no significant differences in the number of visits to the female or male compartments (data not shown). Ultimately, the total time spent investigating both conspecifics was significantly greater with CNO compared to vehicle (Figure 2C), which may indicate increased sociability. Genital grooming episodes during the preference test were similar between vehicle and CNO (Figure 2D).

### 3.3 Effect of CNO on sexual behaviour

Activation of the hM3Dq DREADD receptor resulted in no significant difference in all the components of sexual behaviour examined; mount, intromission and ejaculation latencies were similar with or without CNO treatment and there were no significant differences in mount and intromission frequencies with activation of MePD Kiss1 neurones compared to control (Table 1). The behavioural data in this test category for mice with misplaced injection sites were not significantly different (data not shown).

**Table 1:** Male sexual behaviour following vehicle or clozapine‐*N*‐oxide (CNO) administration

	Mount latency	Intromission latency	Ejaculation latency	Mount frequency	Intromission frequency
Vehicle	918.7 ± 365.0	1080.8 ± 306.0	1421.8 ± 222.9	7.0 ± 4.3	4.2 ± 3.1
CNO	957.3 ± 350.6	959.0 ± 350.0	1109.2 ± 291.1	3.7 ± 2.0	1.6 ± 0.8

Values are expressed as the mean ± SEM, n = 9. Latencies are expressed in seconds.

### 3.4 Effect of CNO on anxiety‐like behaviour and social interaction

**Figure 3:**
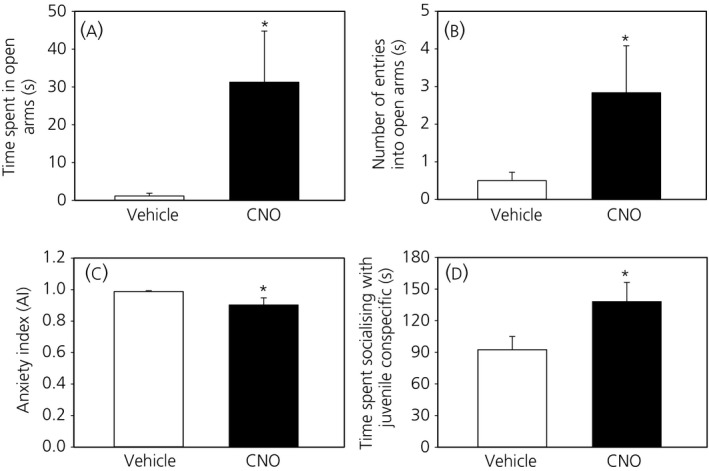
Effect of clozapine‐*N*‐oxide (CNO) on anxiety and social interaction. CNO (5 mg kg^‐1^; i.p.) significantly increased the time spent in the open arms of the elevated plus maze (EPM) (A) and the number of entries into the EPM open arms (B), resulting in a significant reduction in the anxiety index (C). Time spent in social interaction with juvenile conspecific significantly increased following CNO administration compared to vehicle (D). *P < .05 vs vehicle (n = 9). The results are the mean ± SEM

Treatment with CNO resulted in a significant increase in time spent on open arms of the EPM, as well as the number of visits to the EPM open arms (*P* < .05) (Figure 3A,B). Consequently, CNO‐induced DREADD activation evoked a significant reduction in AI in the mice compared to controls (Figure 3C), although not in mice with misplaced injections (AI: CNO; 0.99 ± 0.007 vs Saline; 0.98 ± 0.011). Similarly, the amount of time spent interacting with the juvenile conspecific was significantly increased following CNO administration compared to vehicle (Figure 3D) but similar in mice with misplaced injection sites (CNO; 91.3 ± 12.17 vs Saline; 87.0 ± 7.57 seconds).

## 4 DISCUSSION

The present study utilised a chemogenetic approach via expression of stimulatory DREADDs (hM_3_Dq) to investigate the role of kisspeptin in the MePD on socio‐sexual behaviours in mice. The results obtained show that DREADD‐mediated activation of MePD kisspeptin neurones remarkably enhances the male mice preference for oestrous females and also increases EPM open arm exploration, as well as social interaction, without any effect on sexual behaviour. The strategy of Cre‐dependent DREADDs along with neurone‐specific Cre mice has proved successful in selective neuronal activation,^39^ drawing its strength from its specificity on targeted neuronal cell types by limiting DREADD expression to the Cre location.^30^ The localisation of mCherry red florescence protein in the MePD may serve as a proxy for kisspeptin neurones and its pattern of distribution is remarkably similar to our dtomato expression in the MePD, as reported previously.^26^ Moreover, the expression of hM_3_Dq receptors in neural sites has no constitutive effect on baseline behaviours in the absence of CNO, nor does CNO produce any untoward behavioural effects in control animals.^40^ Cautionary to the role of CNO on behavioural response in chemogenetic studies, a recent study by MacLaren et al^41^ demonstrated that CNO may modulate some behavioural outcome in rats such as reduction in startle response to loud acoustic stimuli, as well as amphetamine‐induced locomotion, necessitating the need for an appropriate CNO‐control group in chemogenetic experiments; the lack of such a group is a limitation of the present study, although the data from mice with misplaced injection sites suggest that CNO may have no underlying effect on the behavioural outcome of our study. Furthermore, CNO treatment in the absence of neural DREADD‐infection did not disrupt the investigation of chemosensory stimuli or receptive sexual behaviour in female mice,^42^ which potentially should be similar in males. Additionally, the premise of data interpretation rests on CNO‐induced activation of kisspeptin‐expressing neurones in the MePD. It is possible that signals expressed in these neurones other than kisspeptin may confer some or all of the noted behavioural events. Additional studies are required to determine whether the observed effects are a result of kisspeptin or another signalling factor co‐released from these kisspeptin‐expressing neurones.

Male mice are characteristically attracted to a sexually receptive female and this approach behaviour is induced by female pheromones.^43^ The results from the present study demonstrate a site‐specific regulatory role for kisspeptin in the motivation to approach an oestrous female. This finding is in close agreement with studies involving *Kiss1r* knockout mice, where male mice displayed equal preference for both male and female conspecifics.^20^ Previous studies have also shown that pheromonal cues conveyed via the accessory olfactory bulb (AOB) induce Fos activation in the MePD of male mandarin voles,^44^ as well as hypothalamic kisspeptin neurones of female mice.^45^ The evidence for reciprocal synaptic innervation between AOB and MePD kisspeptin neurones^46^ lends support to the suggestion that kisspeptin neurones in the MePD do indeed participate in regulating pheromone‐induced sexual partner preference as shown in the present study. The olfactory‐mediated response to the opposite sex is also conserved in humans. Several studies have shown that body odours of women taken around the time of ovulation are more pleasant and attractive to men than those collected at other times of the menstrual cycle,^47^, ^48^ and that this exposure to body odour is associated with mating motivation.^49^ Furthermore, mate odour preference is associated with reward‐seeking activities^50^ and is used as a measure of pleasurable behaviour in male mice.^51^ Female odour activates reward centres in the brain, causing dopamine release from the nucleus accumbens of male mice.^50^ Interestingly, dopaminergic neurones from the reward circuitry form close apposition with kisspeptin neurones in the MePD of male mice.^46^ A synergistic neural pathway involving MePD kisspeptin‐dopamine neurones may therefore mediate the potentiation of sexual partner preference. Testosterone exerts an activational effect on sexual partner preference;^52^ however, it may be limited in the absence of kisspeptin signalling because testosterone priming could not reverse the lack of preference for female odour in *Kiss1r* knockout male mice.^20^ In men, kisspeptin‐induced enhancement of limbic brain activation in response to sexual images is independent of testosterone secretion.^25^ We suggest that the enhanced sexual partner preference observed in the present study may derive from kisspeptin synaptic control rather than from any androgenic influence.

Sexual partner preference positively correlates with copulatory behaviour in rodents. A deficit in sexual partner preference is usually accompanied by prolonged latency to mount or ejaculate.^15^, ^53^ The outcome of enhanced preference score in the present study was, however, not facilitatory to the expression of male sexual behaviour. In male rats, Fos immunoreactivity indicated that a cluster of neurones in the MePD is involved in modulating copulatory behaviour.^54^ Because the levels of male sexual behaviour (mount, intromission and ejaculation) were equivalent in the presence and absence of DREADDs activation of MePD kisspeptin neurones, it is plausible to attribute a limited role for kisspeptin in the MePD on male coital behaviour or to assume that sexual partner preference and sexual behaviour are differentially regulated. We have recently reported a dose‐dependent effect of kisspeptin in the MePD on ex‐copula erections in rats, which is analogous to human psychogenic erections caused by erotica.^21^ Given that the previously utilised pharmacological approach may involve a mechanistic pathway distinct from the DREADDs technique, it is possibile that the endogenous kisspeptin signalling induced by hM_3_Dq receptor activation over a short term may not be sufficient to elicit any change in sexual behaviour.

Furthermore, the MePD is also considered as a neural hub that controls anxiety and social behaviour.^16^, ^18^ Social interaction inversely correlates with anxiety^55^ with decreased social interaction being used as a measure of anxiogenic state. Both increased social interaction and EPM open arm entry have been interpreted as a reduction in anxiety‐like behaviour.^56^ The present study suggests that excitation of MePD kisspeptin neurones via hM_3_Dq activation^57^ elicits neural signals that dampen anxiety. In zebrafish, habenula kisspeptin modulated the fear response and increased exploratory behaviour in a novel tank test.^22^ Similarly, i.c.v. infusion of kisspeptin induced antidepressant‐like effect during a forced‐swim test.^23^ By contrast, i.c.v. injection of kisspeptin resulted in anxiety‐like behaviour in rats.^24^ Differences in the experimental paradigm and species may account for the inconsistencies on the role of kisspeptin in anxiety‐like behaviour. Further studies are warranted to clarify the role of kisspeptin on anxiety.

There is a parallel interaction between anxiety and sexual behaviour. Anxious male mice exhibit reduced sexual motivation^58^ and treatment with anxiolytic agents exerts a corrective effect on sexual interest in depressed mice.^59^ In men, anxiety‐related disorders occur in tandem with sexual dysfunction.^60^ It is therefore not surprising that kisspeptin may coordinate sexual preference and anxiety behaviour in an integrated fashion that is positive towards copulation. Moreover, the enhancement of limbic brain activity by kisspeptin in men viewing sexual images correlates with the attenuation of negative mood and reduced sexual aversion,^25^ which indicates that, by modulating limbic brain activity, kisspeptin is critical for normal reproductive behaviour.

The sexual dimorphic nature of kisspeptin in the MePD could not be explored in the present study because we only investigated the socio‐sexual response of male mice without a female. This limited scope narrows the far‐reaching significance of the present study, although our planned future work aims to ascertain the behavioural outcome in the female. Speculatively, a similar trend may be obtainable in the female, given the response of hypothalamic kisspeptin neurones of female mice to olfactory cues,^45^ in addition to the results of chemogenetic studies in mice showing the involvement of medial amygdala neurones in female sexual behaviour.^42^ Another major limitation of the present study is the inability to characterise in specific details the viral transfection of kisspeptin neurones in the MePD and, consequently, there is lack of empirical evidence on the percentage of kisspeptin neurones activated by CNO. The technical challenge that we experienced using immunohistochemistry, which has a similar occurrence in other studies,^61^ hampered the identification of kisspeptin immunoreactive cells in the MePD. However, the number of positive neurones expressing mCherry fluorescent protein may be indicative of kisspeptin neurones and was comparable to the number of MePD kisspeptin cells previously reported in pubertal male mice,^62^ although it did not reach levels shown in adult males.^19^, ^61^ This disparity may be related to differences in the transgenic mouse line or experimental protocol such as use of in situ hybridisation technique^19^, ^61^ compared to our fluorescence. Moreover, in comparison with female Kiss‐Cre mice,^26^ the number of positive neurones in the MePD of males in the present study was greater and supports the sex variation in MePD kisspeptin population.^19^ However, differences in tissue preparation, the number of sections counted and section thickness cannot be ignored as variable factors.^26^ Future studies are required to address this technical ambiguity.

In conclusion, the data obtained in the present study suggest that activation of kisspeptin neurones in the MePD synchronises socio‐sexual behaviour by enhancing preference for a sexual partner and the anxiolytic response to promote maximal reproductive success in the male.
